# Correlation Between Thrombus Signal Intensity and Aneurysm Wall Thickness in Partially Thrombosed Intracranial Aneurysms Using 7T Magnetization-Prepared Rapid Acquisition Gradient Echo Magnetic Resonance Imaging

**DOI:** 10.3389/fneur.2022.758126

**Published:** 2022-02-18

**Authors:** Taku Sato, Toshinori Matsushige, Bixia Chen, Oliver Gembruch, Philipp Dammann, Ramazan Jabbarli, Michael Forsting, Andreas Junker, Stefan Maderwald, Harald H. Quick, Mark E. Ladd, Ulrich Sure, Karsten H. Wrede

**Affiliations:** ^1^Department of Neurosurgery, University Hospital Essen, University Duisburg-Essen, Essen, Germany; ^2^Erwin L. Hahn Institute for Magnetic Resonance Imaging, University Duisburg-Essen, Essen, Germany; ^3^Department of Neurosurgery, Fukushima Medical University, Fukushima, Japan; ^4^Department of Neurosurgery, Graduate School of Biomedical and Health Sciences, Hiroshima University, Hiroshima, Japan; ^5^Department of Neurosurgery and Interventional Neuroradiology, Hiroshima City Asa Citizens Hospital, Hiroshima, Japan; ^6^Department of Diagnostic and Interventional Radiology and Neuroradiology, University Hospital Essen, University Duisburg-Essen, Essen, Germany; ^7^Institute of Neuropathology, University Hospital Essen, University Duisburg-Essen, Essen, Germany; ^8^High Field and Hybrid MR Imaging, University Hospital Essen, University Duisburg-Essen, Essen, Germany; ^9^Medical Physics in Radiology, German Cancer Research Center (DKFZ), Heidelberg, Germany; ^10^Faculty of Physics and Astronomy and Faculty of Medicine, University of Heidelberg, Heidelberg, Germany

**Keywords:** thrombus, aneurysm wall, partially thrombosed intracranial aneurysm, 7T magnetic resonance imaging, magnetization-prepared rapid acquisition gradient echo

## Abstract

**Objective:**

The objective of this study is to investigate the relationship between the thrombus signal intensity and aneurysm wall thickness in partially thrombosed intracranial aneurysms *in vivo* with magnetization-prepared rapid acquisition gradient echo (MPRAGE) taken using 7T magnetic resonance imaging (MRI) and correlate the findings to wall instability.

**Methods:**

Sixteen partially thrombosed intracranial aneurysms were evaluated using a 7T whole-body MR system with nonenhanced MPRAGE. To normalize the thrombus signal intensity, its highest signal intensity was compared to that of the anterior corpus callosum of the same subject, and the signal intensity ratio was calculated. The correlation between the thrombus signal intensity ratio and the thickness of the aneurysm wall was analyzed. Furthermore, aneurysmal histopathological specimens from six tissue samples were compared with radiological findings to detect any correlation.

**Results:**

The mean thrombus signal intensity ratio was 0.57 (standard error of the mean [SEM] 0.06, range 0.25–1.01). The mean thickness of the aneurysm wall was 1.25 (SEM 0.08, range 0.84–1.55) mm. The thrombus signal intensity ratio significantly correlated with the aneurysm wall thickness (*p* < 0.01). The aneurysm walls with the high thrombus signal intensity ratio were significantly thicker. In histopathological examinations, three patients with a hypointense thrombus had fewer macrophages infiltrating the thrombus and a thin degenerated aneurysmal wall. In contrast, three patients with a hyperintense thrombus had abundant macrophages infiltrating the thrombus.

**Conclusion:**

The thrombus signal intensity ratio in partially thrombosed intracranial aneurysms correlated with aneurysm wall thickness and histologic features, indicating wall instability.

## Introduction

Aneurysmal subarachnoid hemorrhage is a heavy health burden with estimated annual incidence ranges of 6–8 patients per 1,00,000 with high morbidity rates (1). Different diagnostic markers for the instability of the cerebral aneurysm walls were reported (2). Ngoepe et al. mentioned that it is not clear whether thrombosis formation in the aneurysm stabilizes the aneurysm or makes it more likely to rupture (3).

Previous studies have suggested that the pathophysiology of thrombosed intracranial aneurysms differs from that of nonthrombosed aneurysms (4). Most thrombosed aneurysms are large (12–24 mm) or giant (>25 mm) and are complex and associated with a high risk of complications with treatment (5). The overall prevalence of thrombosed intracranial aneurysms remains unknown; however, an autopsy series reported 9% of all intracranial aneurysms (6). Because thrombosed aneurysms are large, surgical treatment is preferred because they are prone to rupture. However, if the aneurysm was stabilized, treatment may not be needed.

The pathophysiology of partially thrombosed aneurysms remains unclear, and only a few studies have performed histological analysis. Atlas et al. have examined partially thrombosed giant aneurysms using 1.5T magnetic resonance imaging (MRI) and showed two histological specimens with macrophages infiltrating the thrombosed aneurysm wall and the peripheral side (7). Martin et al. have evaluated the thrombus characteristics in aneurysms with T1- and T2-weighted imaging using the 1.5T MRI. The thrombus in thrombosed aneurysms was divided into two zones: a core and a rim. The thrombus signal intensity was homogeneous in five patients and heterogeneous in four. The heterogeneous thrombus is characterized by peripheral hyperintensity toward the arterial wall side of the thrombus. The imaging appearance of the thrombus was very stable over long periods; however, they stated that the study was limited due to the lack of histological confirmation (8). A recent evaluation of a thrombosed aneurysm with ultra-high-field MRI examined the associated pathophysiology (9). The study found that aneurysm wall microstructures responsible for gadolinium enhancement are not shown at lower spatial resolutions but could be visualized *in vivo* using high-resolution gadolinium-enhanced 7T MRI. However, no studies have evaluated the correlation between the radiological findings of the thrombus and the aneurysm wall thickness.

Therefore, this study aimed to investigate the correlation between radiological findings of the thrombus and the aneurysm wall thickness in partially thrombosed intracranial aneurysms using magnetization-prepared rapid acquisition gradient echo (MPRAGE) at 7T to confirm wall instability.

## Materials and Methods

### Study Design and Population

Patients were prospectively enrolled between January 2011 and November 2018. The diagnostic study cohort comprised 7 men and 8 women with an average age of 55.7 (range 43–75) years at a single institution. Inclusion criteria were (1) patients with a partially thrombosed intracranial aneurysm diagnosed by digital subtraction angiography and conventional computed tomography or 3T MRI, (2) ≥18 years old, and (3) able to provide informed consent. Exclusion criteria were (1) the presence of a cardiac pacemaker or any other electronic implants, (2) pregnancy or breastfeeding, (3) claustrophobic, or (4) patients requiring permanent monitoring devices (e.g., subarachnoid hemorrhage).

Many thrombosed cerebral aneurysms are large and carry a high risk of complications with treatment. Therefore, clipping an aneurysm is often selected for lesions in relatively shallow positions with a wide surgical field. Still, there is a tendency to choose endovascular treatment for lesions in deep locations such as PCA and ICA. However, in our study, the neuroradiologists and neurosurgeons discussed on a case-by-case manner and together decided the appropriate treatment plan.

### MRI Scanners and Coil Systems

All patients were evaluated using a 7T whole-body MR system (MAGNETOM 7T [prototype model], Siemens Healthcare GmbH, Erlangen, Germany) equipped with a 1/32-channel Tx/Rx head radiofrequency coil (Nova Medical, Wilmington, USA). The gradient system provides 40 mT/m maximum amplitude and a slew rate of 200 mT/m/ms.

### MPRAGE Sequence

A modified MPRAGE sequence was obtained based on the following sequence parameters: field of view = 270 × 236 mm^2^, matrix = 384 × 336, resolution = 0.7 × 0.7 mm^2^, slice thickness = 0.7 mm, repetition time = 2,500 ms, echo time = 1.54 ms, flip angle = 7°, bandwidth = 570 Hz/pixel, and acquisition time = 6 min 13 s (10, 11). Gadolinium contrast enhanced images were acquired 10 min after intravenous administration of a gadobutrol-based macrocyclic contrast agent (1 mmol/ml/10 kg body weight).

### Image Evaluation

Two raters assessed the following characteristics in multiplanar image reconstruction using the Horos™ open-source medical image viewer (Horos Project, Geneva, Switzerland) in consensus reading: (1) thrombus signal intensity, (2) aneurysm wall thickness, and (3) maximum diameter of the aneurysmal dome. A previous report using 7T MPRAGE demonstrated that aneurysm walls were delineated with a mostly heterogeneous hypointense signal. The aneurysm wall enhancement patterns in thrombosed intracranial aneurysms were distinguished as either single or double rim patterns. The signal intensities of the intraluminal thrombus were heterogeneous or homogeneous. The thrombus areas adjacent to the aneurysm wall were generally hyperintense as compared to the wall (12). We drew the three-dimensional volumetric mask in the thrombus of the aneurysm and then measured its average intensity values.

To measure the signal intensity as in a previously described study, the value for all patients included in the study should be normalized. Therefore, the signal intensity of the pathology can be normalized by correlating it to an anatomical control structure, which shows no intensity changes with age. Brambilla et al. reported no significant relationship between age and MRI signal intensity measures in the corpus callosum among healthy patients (13). Accordingly, to normalize the thrombus signal intensity of the aneurysm, the signal intensity ratio of each thrombus was measured by dividing the thrombus signal intensity by the average signal intensity of four voxels in the anterior corpus callosum. The average value of four measurements across the wall was recorded for the wall thickness. The aneurysm walls were depicted as a mostly heterogeneous hypointense signal. We classified the extent of thrombosis into three grades (1= mild, 2 = moderate, and 3 =severe). For each mean value, the standard error of the mean (SEM) was calculated as an estimation of the population mean.

### Histopathological Examination

Histopathological samples of six thrombosed intracranial aneurysms (patients 5, 6, 10, 11, 14, and 15), harvested during the microsurgical clipping of the aneurysm, were analyzed and compared with MRI findings. Histopathologic sections from areas approximately corresponding to MRI ROIs were prepared with 5-μm thickness and stained with hematoxylin and eosin, Verhoeff-Van Gieson, Prussian blue, and a cluster of differentiation (CD) 68. The aneurysm wall thickness was measured in five representative locations, and the mean value and range were calculated. The degree of macrophage infiltration was evaluated using the ImageJ software package (National Institutes of Health, Bethesda, Maryland) (14). First, CD68 immunostainings were digitized using an Aperio AT2 slide scanner (Leica Microsystems, Wetzlar, Germany). Using the scanned files, four images of the thrombus and aneurysm wall with an edge length of 300 μm and the highest macrophage activity covering the thrombus were extracted for further analysis. The CD68-positive areas of all four ROIs were measured and calculated as a percentage using the ImageJ functions “color threshold” and “analyze particles.”

### Statistical Analysis

Spearman's rank correlation coefficient analysis was performed between the signal intensity ratio of the thrombus and the aneurysm wall thickness, age, or aneurysm size variables. A Mann–Whitney *U* test analyzed the correlation between the signal intensity and contrast enhancement patterns. Significance level α was defined as *p* < 0.05.

## Results

**Figure 1:**
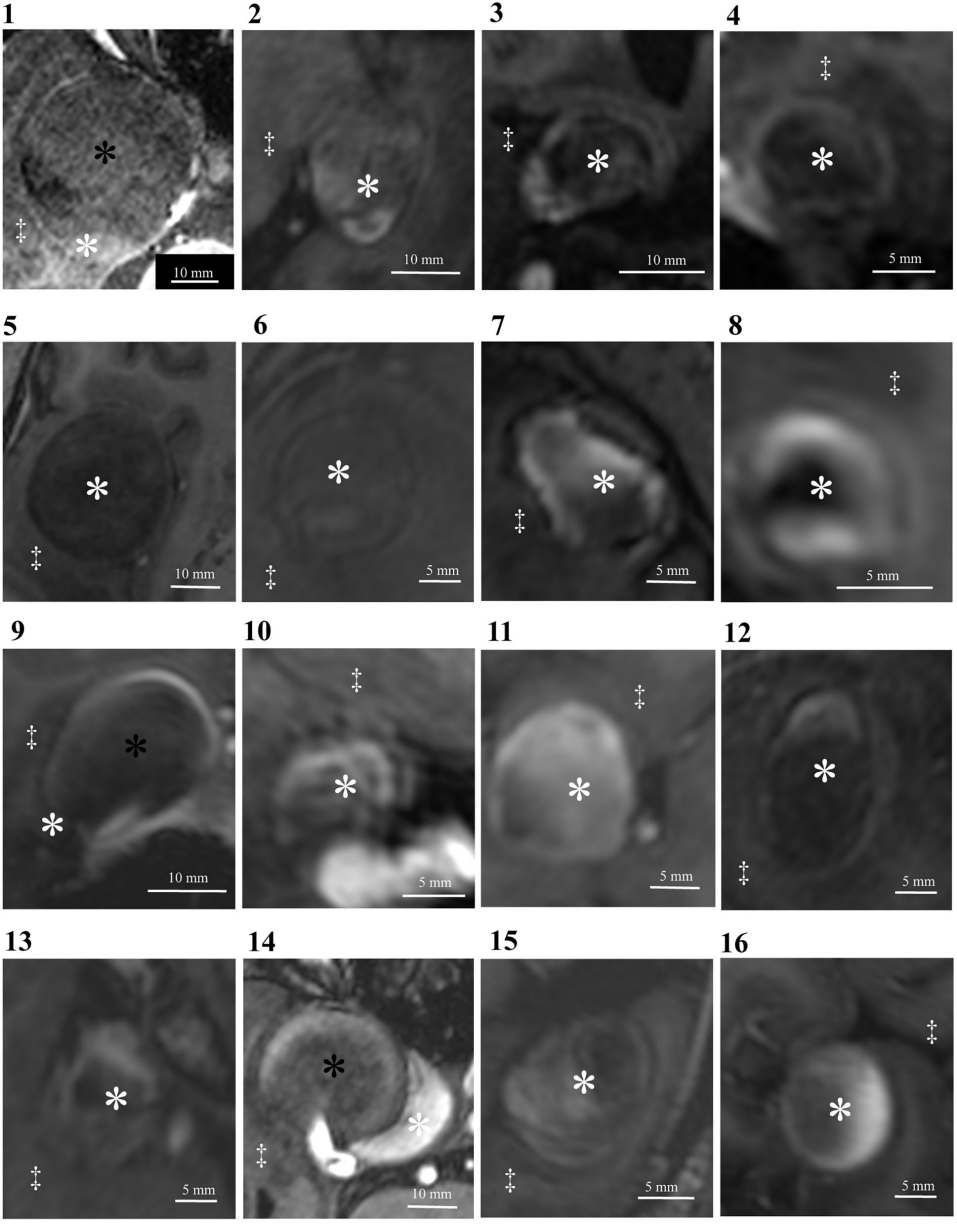
Delineation of thrombosed intracranial aneurysms using 7T MRI. Identical structures are marked in all subfigures as follows. White asterisks: intraluminal thrombus; black asterisks: aneurysm lumen; double daggers: brain parenchyma.

All 15 patients were examined without any adverse events, and all MRI sequences were successfully obtained. Basic demographic data for all patients and major anatomical features of aneurysms are summarized in Table 1. Imaging studies revealed partial thrombosis in 16 aneurysms (Figure 1). Heterogeneous thrombus characteristics exhibited peripheral hyperintensity toward the arterial wall side of the thrombus. The heterogeneous and homogeneous aneurysms were 11 and 5 lesions, respectively. One patient (patient 7) had two aneurysms with partial thrombosis. The average intensity of corpus callosum was 261.75 (SEM 18.8, range 138–420).

**Table 1:** Patient demographics and anatomic characteristics.

**Aneurysm Nr**.	**Patient Nr**.	**Age (year)**	**Sex**	**Location**	**Maximum diameter (mm)**	**Treatment**	**High-intensity ratio**	**Wall thickness (mm)**	**Wall thickness in histology (range) (mm)**
1^a^	1	56	F	ICA	37.9	Endovascular^c^	0.63	1.02	N.A.
2	2	45	M	PCA	20.0	Endovascular^d^	0.64	1.55	N.A.
3	3	69	F	ICA	25.1	Endovascular^c^	0.48	1.52	N.A.
4	4	44	M	dACA	13.2	Clipping	0.37	0.98	N.A.
5^b^	5	75	M	MCA	35.8	Clipping	0.30	0.94	0.92 (0.83–0.95)
6^a, b^	6	61	F	MCA	26.0	Clipping	0.35	0.87	0.72 (0.24–0.80)
7^a^	7	55	M	MCA	25.0	Clipping	0.91	1.88	N.A.
8^a^	7	55	M	dACA	11.0	Clipping	1.01	1.54	N.A.
9^a^	8	52	F	ICA	23.2	Endovascular	0.25	0.92	N.A.
10^a^	9	63	F	ICA	21.4	Endovascular	0.84	1.50	N.A.
11^a^	10	56	M	MCA	29.6	Clipping	0.62	1.53	1.05 (0.94–1.30)
12^a^	11	53	M	MCA	14.2	Clipping	0.26	0.84	0.88 (0.48–1.02)
13^a^	12	70	F	MCA	15.6	Clipping	0.40	0.91	N.A.
14^a^	13	43	M	ICA	36.0	Endovascular^c^	0.88	1.36	N.A.
15^a^	14	50	F	MCA	21.6	Clipping	0.55	1.11	1.13 (0.89–1.21)
16^a^	15	44	F	MCA	28.2	Clipping	0.64	1.55	1.15 (0.76–1.23)
**Aneurysm Nr**.	**Extent of thrombus**	**Wall contrast enhancement pattern**	**Aneurysm shape**	**Type of thrombus signal**	**Past history**	**Anticoagulant**	**Antiplatelet agent**		
1	1	Single	Regular	Homogeneous	No	No	No		
2	3	N.A.	Regular	Heterogeneous	Basedow	No	Aspirin		
3	3	N.A.	Irregular	Heterogeneous	Hypothyroidism	No	No		
4	3	N.A.	Regular	Heterogeneous	Epilepsy	No	No		
5	2	N.A.	Regular	Heterogeneous	Hypertension, PAD	No	No		
6	2	Double	Regular	Homogeneous	COPD, PAD, Hypertension	No	No		
7	2	Double	Irregular	Heterogeneous	Gastritis, Hypertension	No	Aspirin		
8	2	Single	Irregular	Heterogeneous	No	No	No		
9	1	Single	Regular	Homogeneous	No	No	Aspirin		
10	2	Single	Regular	Homogeneous	No	No	No		
11	2	Double	Irregular	Heterogeneous	No	No	No		
12	3	Double	Irregular	Heterogeneous	Epithelial carcinoma, Hypertension	No	No		
13	2	Single	Irregular	Heterogeneous	Migraine	No	No		
14	2	Single	Regular	Homogeneous	No	No	No		
15	3	Single	Irregular	Heterogeneous	Varices, Ovariectomy	No	No		
16	3	Double	Regular	Heterogeneous	Hypertension	No	No		

*dACA indicates distal anterior cerebral artery; N.A., not available*.

a *Aneurysms 1, 6, 7, 8, 9, 10, 11, 12, 13, 14, 15, and 16 have been reported in the context of wall contrast enhancement of thrombosed intracranial aneurysms at 7T MRI*.

b *Aneurysms 5 and 6 have been reported in the context of giant intracranial aneurysms imaged at 7T MRI*.

c *Parent vessel occlusion*.

d *Coiling with a stent*.

*Extent of thrombosis (1, mild; 2, moderate; and 3, severe); N.A., not available; PAD, peripheral arterial disease; COPD, chronic obstructive pulmonary disease. Aspirin (daily 100mg aspirin medication over at least 6 months)*.

The mean diameter of aneurysms was 24.0 (SEM 2.0, range 11.0–37.9) mm. The mean signal intensity ratio of the thrombus was 0.57 (SEM 0.06, range 0.25–1.01). The mean aneurysm wall thickness was 1.25 (SEM 0.08, range 0.84–1.55) mm. Regarding the extent of thrombosis, it was mild in two, moderate in eight, and severe in six cases.

**Figure 2:**
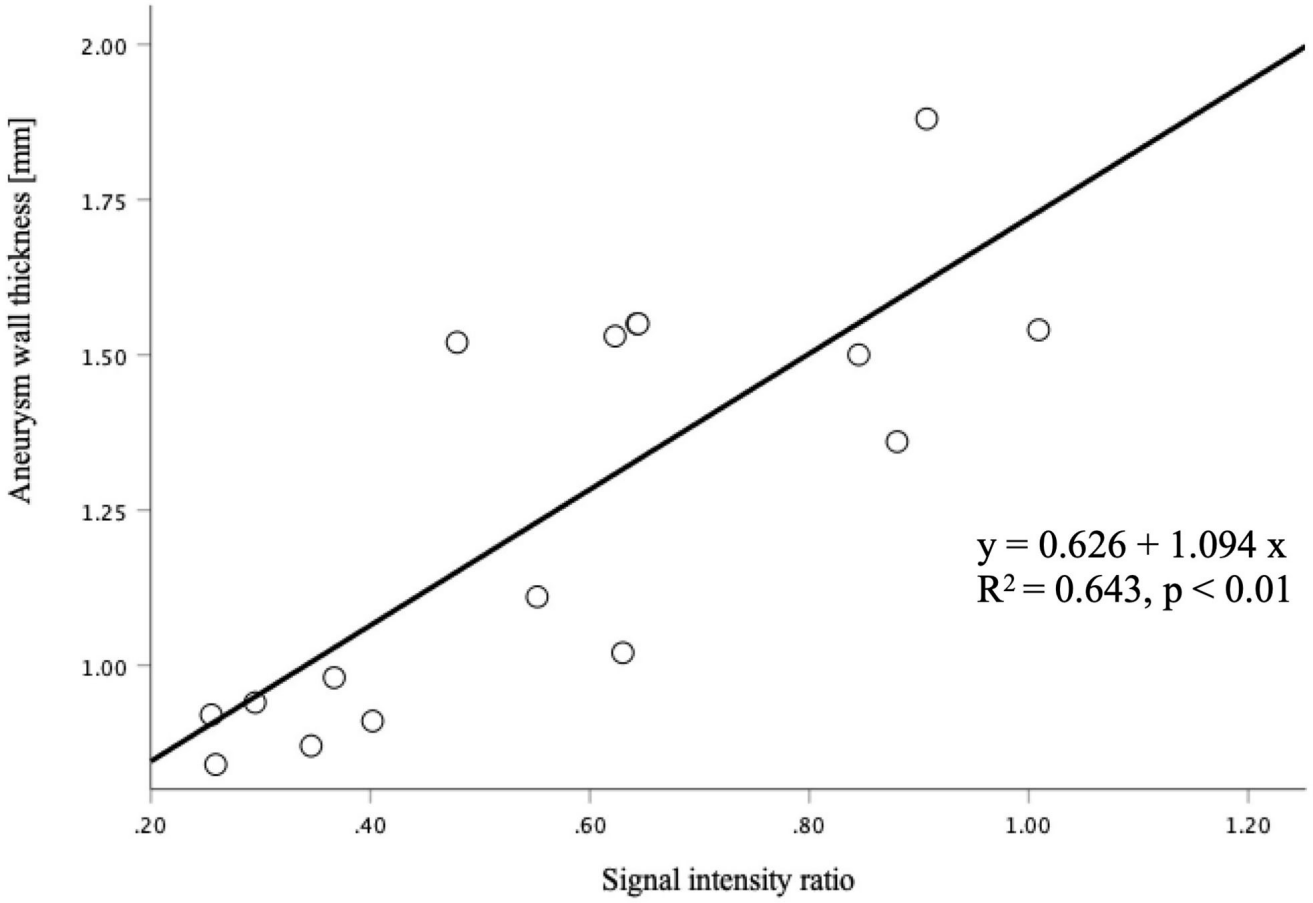
The correlation between the signal intensity ratio of the thrombus and aneurysm wall thickness was statistically significant (*p* < 0.01).

**Figure 3:**
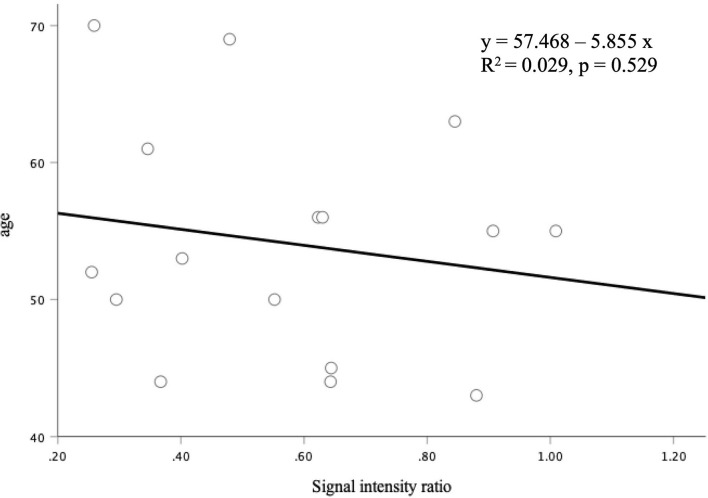
The correlation between the signal intensity ratio of the thrombus and age was not statistically significant (*p* = 0.529).

**Figure 4:**
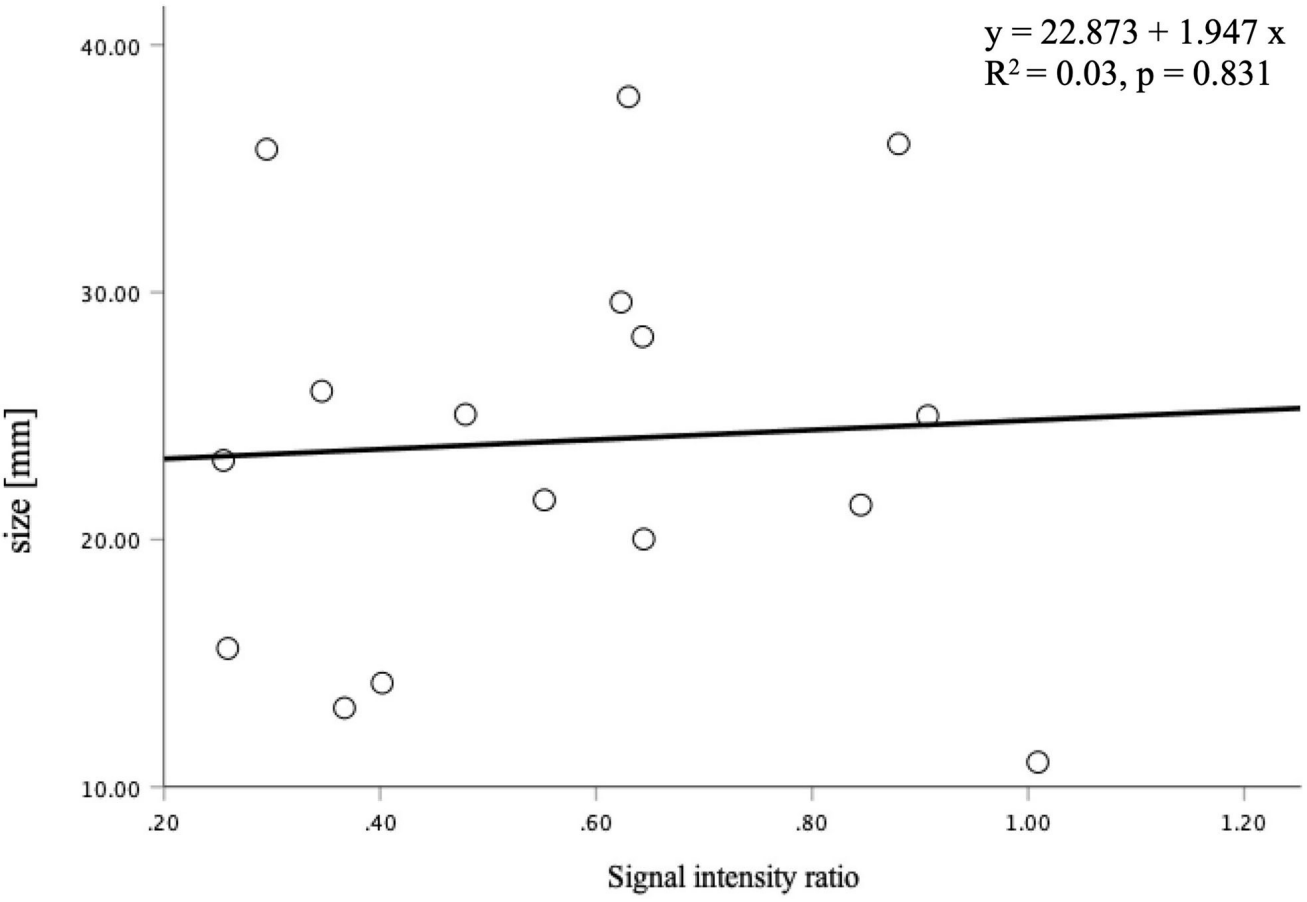
The correlation between the signal intensity ratio of the thrombus and aneurysm size was not statistically significant (*p* = 0.831).

The signal intensity ratio of the thrombus significantly correlated with the aneurysm wall thickness (*p* < 0.01) (Figure 2). The aneurysm walls with a higher signal intensity ratio of the thrombus were thicker, which did not correlate with age (*p* = 0.529) (Figure 3) or aneurysm size (*p* = 0.831) (Figure 4). Except for the not available data, no correlation was between the signal intensity and contrast enhancement patterns (*p* = 0.44).

**Figure 5:**
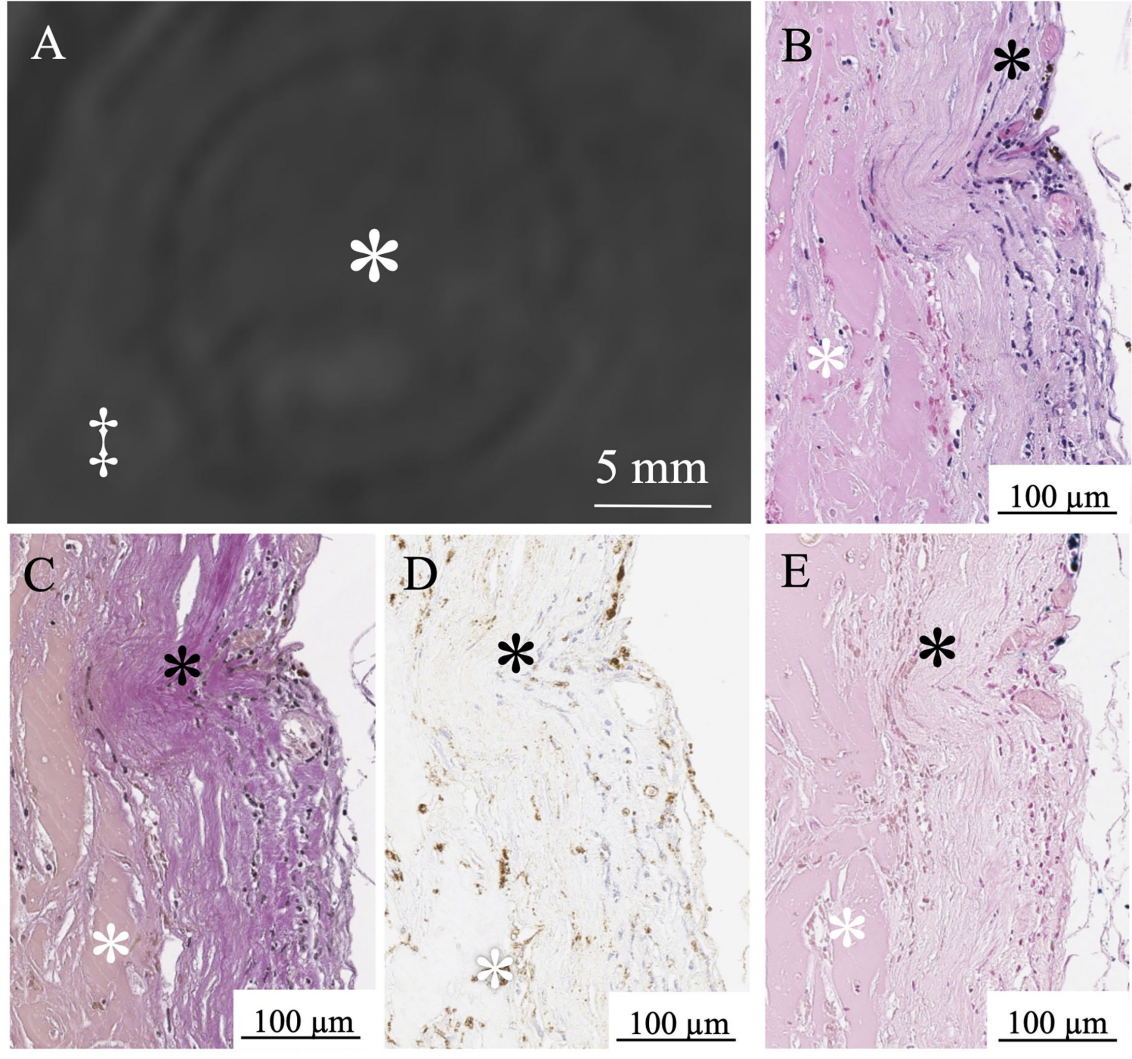
MPRAGE shows a low signal intensity thrombus in Aneurysm No. 6 (white asterisk: intraluminal thrombus; double daggers: brain parenchyma) **(A)**. White asterisk indicates intraluminal thrombus. A few red blood cells in the thrombus (white asterisk) and thin aneurysm wall (black asterisk) in H&E staining **(B)**. Loss of elastic fibers in the aneurysm wall layer in Verhoeff-Van Gieson staining (black asterisk) **(C)**. A few macrophages (white asterisk) in the thrombus in CD68 immunostaining **(D)**. Limited iron deposition (white asterisk) in the thrombus stained with Prussian blue **(E)**.

**Figure 6:**
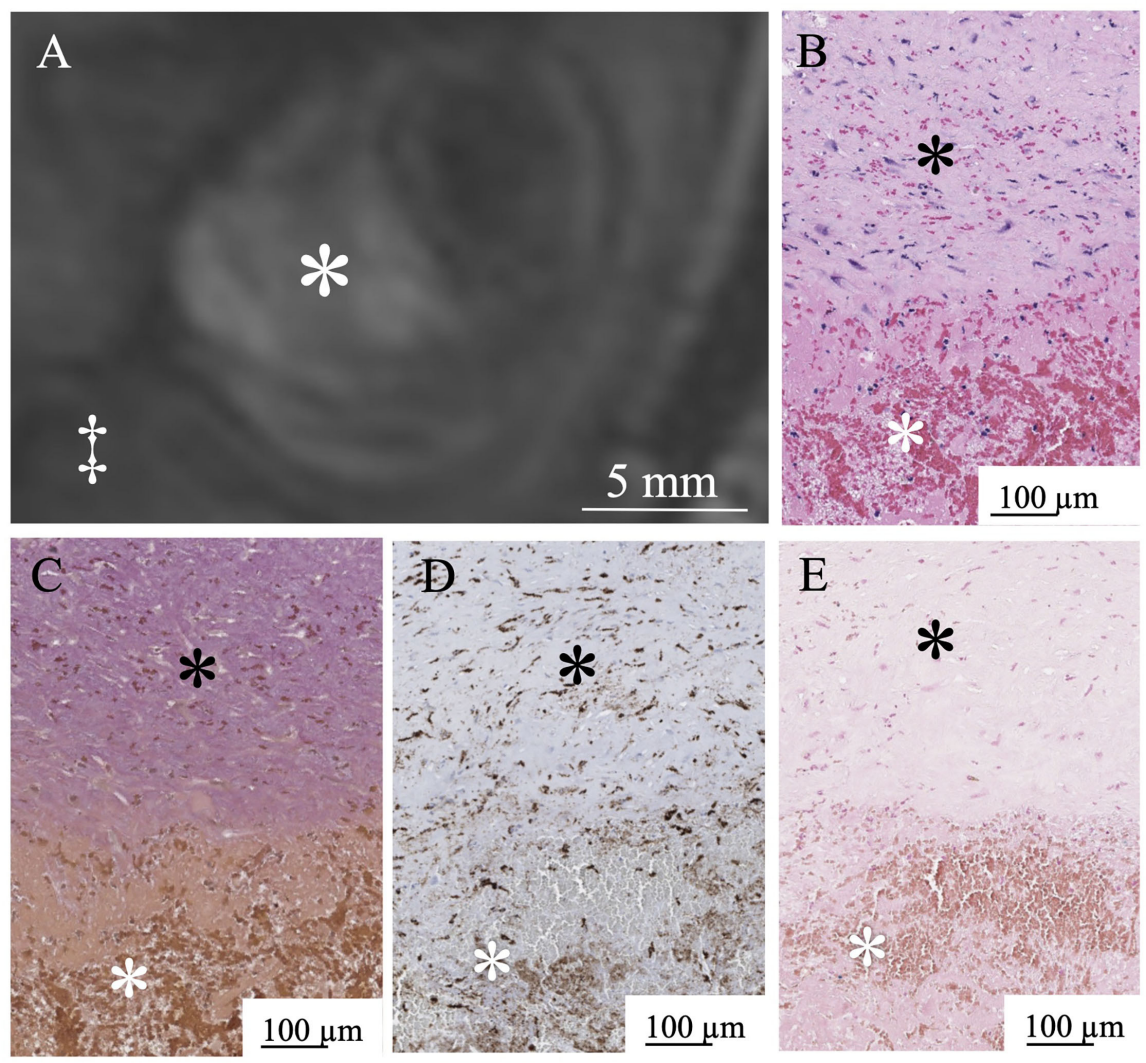
MPRAGE shows a high signal intensity pattern in Aneurysm No. 15 (white asterisk: intraluminal thrombus; double daggers: brain parenchyma) **(A)**. White asterisk indicates intraluminal thrombus. Abundant red blood cells in the thrombus (white asterisk) in H&E staining **(B)**. Thick aneurysm wall layer in the Verhoeff-Van Gieson staining (black asterisk) **(C)**. Abundant macrophages (white asterisk) in the thrombus in CD68 immunostaining **(D)**. Limited iron deposition (white asterisk) in the thrombus stained with Prussian blue **(E)**.

In six aneurysms, partial resection of the aneurysm dome was required during the microsurgical procedure to expose the neck, and these specimens were suitable for histological examination. In histopathological examinations, three patients with a thrombus signal intensity ratio of < 0.55 in MPRAGE included a few macrophages in the thrombus and a thin aneurysmal wall (Figure 5). Conversely, three patients with a thrombus signal intensity ratio of ≥0.55 in the MPRAGE had thick aneurysm walls and numerous thrombus macrophages (Figure 6). The mean aneurysm wall thicknesses (aneurysms 5, 6, 11, 12, 15, and 16) in the histological examination were 0.92 (range 0.83–0.95), 0.72 (0.24–0.80), 1.05 (0.94–1.30), 0.88 (0.48–1.02), 1.13 (0.89–1.21), and 1.15 (0.76–1.23) mm, respectively. The average CD68-positive areas in the high signal intensity thrombus were larger (40.1 ± 27.6%) than those with low signal intensity with a CD68-positive area (5.4 ± 5.1%). CD68-positive area in the aneurysm wall was 15.4 ± 3.4%.

## Discussion

In this study, we found that the signal intensity ratio of the thrombus in partially thrombosed aneurysms is significantly positively correlated with the aneurysm wall thickness.

The mechanism of thrombus occurrence and development in a thrombosed aneurysm is unknown. Vorkapic et al. have reported that the thrombosed part has onion-like layers, each with different signal intensities. A low signal in the center and a high signal in the peripheral side were highly suggestive of a partially thrombosed aneurysm. Such aneurysms showed layers of fresh thrombus within the peripheral side, whereas older thrombus was present in the central parts (15). A study using ultra-high-field MRI is required to confirm it. Obusez et al. have shown that the primary clinical advantages of 7T magnets, including higher signal-to-noise ratio, higher contrast-to-noise ratio, smaller voxels, and stronger susceptibility contrast, may increase lesion conspicuity, detection, and characterization compared to low field 1.5T and 3T (16). Harteveld et al. have shown that vessel wall visibility was equal or significantly better at 7T than 3T for the studied arterial segments (17). They also mentioned most vessel wall lesions in the cerebral arteries can be displayed using 7T MRI. Recently, *in vivo* visualization using high-resolution gadolinium-enhanced 7T MRI showed two distinct aneurysm wall microstructures in the thrombosed aneurysm wall. The inner wall enhancement correlated with neovascularization of the inner wall layer and the adjacent thrombus, whereas the outer wall enhancement correlated with the formation of vasa vasorum in the outer aneurysm wall layer. These microstructures are responsible for distinct gadolinium enhancement patterns not depicted at the low spatial resolution. The enhancement could be demonstrated to be relevant for understanding the histopathology of aneurysm wall inflammation (9). However, no relationship between the thrombus and the aneurysm wall has been evaluated.

Previous reports have shown that 7T MRI could assess the aneurysm wall thickness (12, 18). Kleinloog et al. have found correlations between variations in MRI signal intensity with variations in the actual aneurysm wall thickness. *Ex vivo* MRI showing variation in signal intensity across the wall of two biopsies was similar to that observed in the *in vivo* images (18). In this study, the relationship between the signal intensity ratio of the thrombus and aneurysm wall thickness was evaluated. The signal intensity ratio of the thrombus significantly correlated with the aneurysm wall thickness (*p* < 0.01). The aneurysm walls with a lower signal intensity ratio of the thrombus were significantly thinner. In our patients, the signal intensity of the thrombus was determined to be either homogeneous or heterogeneous in MPRAGE at 7T MRI, and heterogeneous thrombus exhibited peripheral hyperintensity toward the arterial wall side of the thrombus as previously reported (8). Although the relationship between the aneurysm wall thickness and the aneurysmal rupture remains unknown, the signal intensity ratio of the thrombus might serve as an *in vivo* biomarker. Assessment of partially thrombosed aneurysms can be a promising clinical application filed for ultra-high-field MRI.

The signal intensity ratio of the thrombus did not correlate with age or aneurysm size in our study. The occurrence of thrombosed intracranial aneurysms might be age-dependent. Even if the thrombosed aneurysm was growing, the signal intensity ratio of the thrombus might be changed over time in several ways. Also, the extent of thrombus did not correlate with other variables (e.g., age, aneurysm size, and high-intensity ratio). This is possibly due to the low number of cases. The imaging appearance of the thrombus was stable over long periods in a previous report (8); however, the follow-up duration in this report was approximately 10.3 months only. Although thrombosed aneurysms need long-term follow-ups, they would need to be treated surgically because their aneurysm size is generally large. Therefore, thrombosed aneurysms cannot be followed up for long periods.

According to our histological findings, a lower signal intensity ratio of the thrombus in MPRAGE is associated with a thin aneurysmal wall. In contrast, a higher signal intensity ratio of the thrombus may indicate remodeling activity of the thrombus. Ollikainen et al. have shown that aneurysm wall remodeling and histologic findings suggest instability and are associated with chronic inflammation histopathologically (19). Lack of internal elastic lamina, erosion of luminal endothelium, infiltration of inflammatory cells, apoptosis of smooth muscle cells, and the presence of myointimal hyperplasia, fibrosis, and thrombus are the characteristics of aneurysm wall remodeling. Marbacher and Frösen et al. demonstrated that continuous exposure of the unorganized thrombus to the circulation can promote neutrophil and inflammatory cell recruitments in experimental models. The signal intensity ratio of the thrombus in this study may correlate with histologic findings suggestive of aneurysm wall instability; however, the pathophysiology of the thrombus and correlative instability in the aneurysm wall is still not completely understood (20, 21). We examined the histological findings in only six aneurysms, and we found the wall thickness in each specimen was approximately close to the thickness measured in the MRI (Table 1). We cannot statistically analyze the correlation between the wall thickness of the specimens and MRI due to the small sample size; however, the continuous accumulation of aneurysmal wall samples henceforth can help us confirm this correlation statistically in the future.

This study has some limitations that should be mentioned. The study cohort only comprised 15 patients with 16 partially thrombosed intracranial aneurysms. This is mainly due to the low prevalence of thrombosed intracranial aneurysms and the even lower rate of unruptured thrombosed intracranial aneurysms. The vessel wall is very thin, there is a problem with the resolution of MRI, and there is a limit to the measurement of the thickness of the blood vessel wall. Furthermore, we did not include other MRI sequences in the measurement of the thrombus signal and wall thickness (e.g., T1WI, T2WI, T2^*^ mapping, and Black Blood) (22, 23). Also, histopathological correlations were only possible in the six aneurysms we studied. The pathological specimen is only a part of the excised aneurysm and not the whole wall of the aneurysm. Unfortunately, we cannot confirm the anatomical correspondence between these specimens and their anatomical locations in the MRI.

In conclusion, the signal intensity ratio of the thrombus in partially thrombosed intracranial aneurysms correlates with aneurysm wall thickness and histologic features, which indicates wall instability at 7T MPRAGE MRI.

## Data Availability Statement

The MRI and personal data cannot be shared publicly. Subjects were recruited and informed written consent was obtained, with Ethical approval from the authorized Ethical Committee of the local university. The local Ethics Committee does not directly regulate access to data and should not be approached about such issues. Data access lies under the overall governance of the sponsoring organization and ultimately depends on what the subjects have consented to. Requests to access the datasets should be directed to Karsten H. Wrede, Karsten.Wrede@uk-essen.de.

## Ethics Statement

The studies involving human participants were reviewed and approved by Untersuchungen mit Magnetresonanztomographie bei 7 Tesla and the Ethics Commission of the Medical Faculty of the University Duisburg-Essen. The patients/participants provided their written informed consent to participate in this study.

## Author Contributions

TS, TM, BC, OG, PD, RJ, MF, AJ, SM, ML, HQ, US, and KW helped in conception and design, or acquisition of data, or analysis and interpretation of data, involved in drafting the article or revising it critically for important intellectual content, contributed in final approval of the version to be published, and carried out the agreement to be accountable for all aspects of the work in ensuring that questions related to the accuracy or integrity of any part of the work are appropriately investigated and resolved. All authors contributed to the article and approved the submitted version.

## Funding

This study has received funding from the University Duisburg-Essen (IFORES grant: Programm zur internen Forschungsförderung Essen) (grant numbers: D/107–40430, D/107–40770, and D/107–40960) and a research grant from the Japanese Society of Neuroradiology.

## Conflict of Interest

The authors declare that the research was conducted in the absence of any commercial or financial relationships that could be construed as a potential conflict of interest.

## Publisher's Note

All claims expressed in this article are solely those of the authors and do not necessarily represent those of their affiliated organizations, or those of the publisher, the editors and the reviewers. Any product that may be evaluated in this article, or claim that may be made by its manufacturer, is not guaranteed or endorsed by the publisher.
